# Self-restricted circular RNA circSOX2 suppressed the malignant progression in SOX2-amplified LUSC

**DOI:** 10.1038/s41419-022-05288-5

**Published:** 2022-10-15

**Authors:** Yingkuan Liang, Nan Wang, Yijian Zhang, Wei Jiang, Chen Fang, Yu Feng, Haitao Ma, Feng Jiang, Gaochao Dong

**Affiliations:** 1 Department of Thoracic Surgery, the First Affiliated Hospital of Soochow University, 215006 Suzhou, China; 2 Department of Thoracic Surgery, Dushu Lake Hospital Affiliated to Soochow University, Medical Center of Soochow University, Suzhou Dushu Lake Hospital, 215000 Suzhou, China; 3 Department of Thoracic Surgery, Nanjing Medical University Affiliated Cancer Hospital, Jiangsu Key Laboratory of Molecular and Translational Cancer Research, Cancer Institute of Jiangsu Province, 210009 Nanjing, China

**Keywords:** Non-small-cell lung cancer, Prognostic markers

## Abstract

Lung squamous cell carcinoma (LUSC) is a histological subtype of non-small cell lung cancer with the worse progression. SRY-Box Transcription Factor 2 (SOX2) copy number amplification (CNA) is the oncogenic driver in ~60% of patients diagnosed with LUSC. Thus, *SOX2* represents an effective therapeutic target in *SOX2*-amplified LUSC. However, SOX2 protein was considered undruggable. Here, we report the expression of a circular RNA, cicSOX2 in *SOX2*-amplified LUSC. Patients with *SOX2*-CAN LUSC expressing circSOX2 manifested increased survival outcomes. CircSOX2 suppressed the proliferation, metastasis, and sphere formation in *SOX2*-amplified LUSC in vitro and in vivo. CircSOX2 originates in the reverse strand of the *SOX2* gene and its sequence was reverse complement to partial 3’UTR of SOX2-coding transcript (mSOX2). CircSOX2 bound to AUF1 and occupied in the 3’UTR of mSOX2, inducing the degradation of mSOX2. In general, circSOX2 is an endogenous self-restricted circRNA in *SOX2*-amplified LUSC. CircSOX2 might be an effective and stable nucleic acid drug candidate in *SOX2*-amplified LUSC with low immunogenicity.

## Introduction

Lung cancer is the most lethal malignancy worldwide, causing 1.7 million deaths each year [1]. Non-small cell lung cancer (NSCLC) accounts for ~85% of lung cancer, and lung squamous cell carcinoma (LUSC) is the usual histologic subtype identified in 25–30% of cases [2]. Patients with LUSC tend to be older and diagnosed at an advanced stage, with a high incidence of co-morbidities. LUSC generally originates in the central proximal bronchi, which increases the likelihood of an attack on larger blood vessels [3]. Therefore, the median survival of patients with LUSC is almost 30% shorter than in other types of NSCLC [4]. SRY-box transcription factor 2 (SOX2) is the functional lineage-survival oncogene in LUSC. SOX2 is located on chromosome 3q26-qter, and this region has been copy number amplified (CNA) in 40–60% of cases with LUSC [5, 6]. The dysregulated expression of SOX2 results in tumorigenesis and therapy resistance in various cancer types [7]. SOX2 appears to activate a fundamental mechanism that allows cancer cells to acquire a stem-like phenotype regardless of the original lineage of cancer cells [8]. Nonetheless, the SOX2 protein plays a critical role as a transcription factor during the progression from normal bronchial epithelium to squamous cell carcinoma by increasing the expression of markers of squamous histology such as P63 and cytokeratin 6A [9]. Nevertheless, SOX2 protein has been considered undruggable. The use of several target site interventions, such as chromatin modifiers, peptide aptamers, SOX2 peptide immunization, small molecule inhibitors of the signaling system, and the PROteolysis Targeting Chimera (PROTAC) cause direct degradation of SOX2 [10].

Circular RNAs (circRNAs) are non-coding RNAs, which are distinguished by the absence of 5′ N7-methylguanosine (m7G) caps and 3′ polyadenylated tails, as well as the presence of covalently closed single-stranded structure [11]. Compared with other coding or non-coding RNAs, circRNA is highly stable, with a four times longer half-life than cognate liner RNAs [12] in the cell, blood, urine, and saliva [13]. Thus, circRNA was supposed to be an effective biomarker and nucleic acid drug [14, 15]. Numerous studies have reported the role of circRNA in almost every activity of life mediated by protein sponging. CircPOK increased the IL6 transcript by combining with ILF2/3 to occupy the promoter of IL6 [16]; The circIMMP2L regulated epigenetic remolding in esophageal squamous cell carcinoma by inducing CtBP1 nucleus retention [17]; The circACC1 up-regulated the glycolysis and β-oxidation by ensuring the stability of AMP-activated protein kinase (AMPK) protein [18]; The circVAMP3 negatively regulated hepatocellular carcinoma (HCC) mediated by cell cycle-associated protein 1 (CAPRIN1) and Stress Granule Assembly Factor 1 (G3BP1) interaction-dependent phase separation [19]. Some nuclear-localized circRNAs played roles in the splicing and transcription of its cognate endogenous transcript [20, 21]. CircSEP3, which was localized in the nucleus, was bound tightly to its parental DNA to form the circRNA–DNA hybrid, resulting in transcriptional pause [22]. Exon–intron circRNAs (EIciRNAs) such as circEIF3J and circPAIP2 regulated their parental mRNA transcription by interacting with U1 snRNA in the nucleus [21]. However, the circRNA derived from the *SOX2* gene has yet to be reported.

AU-rich element rna-binding protein 1 (AUF1) is an RNA-binding protein that recognizes AU-rich sequences in the 3’-untranslated regions (3’UTR) region of mRNA and regulates its stability and/or translation [23]. In DICER1 syndrome, AUF1 competed with PUMILIO for binding to the DICER1 mutation allele, thereby degrading DICER1 mRNA [24]. CircPCNX interacted with AUF1 carrying the “AUUAACUUU” element to retain the expression of P21 mRNA [25]. AUF1 interacted with the 3’UTR of *MYC* mRNA to promote its translation in chronic myelogenous leukemia [26]. However, the role of AUF1 in circRNA regulation remains to be investigated.

In our study, we reported a SOX2-amplified associated circRNA, circSOX2, which was driven from the reverse strand of the SOX2 gene and effectively expressed in SOX2-applied LUSC cells. The SOX2-CNA LUSC patients with higher circSOX2 expressed better survival. CircSOX2 suppressed the proliferative, metastasis, and sphere-forming in LUSC with SOX2 amplification in vitro and in vivo. The sequence of circSOX2 was a reverse complement to the part 3’ UTR of the SOX2 coding transcript (mSOX2). The nuclear predominate circSOX2 promoted the SOX2 coding transcript degradation by sponging and guiding AUF1 to the 3’ UTR of the SOX2 coding transcript. Thus, circSOX2 was a functional self-restriction circRNA for SOX2 amplification and an effective nucleic acid drug candidate for SOX2-amplified LUSC.

## Results

### CircSOX2 is efficiently expressed in SOX2-amplified LUSC

**Fig. 1:**
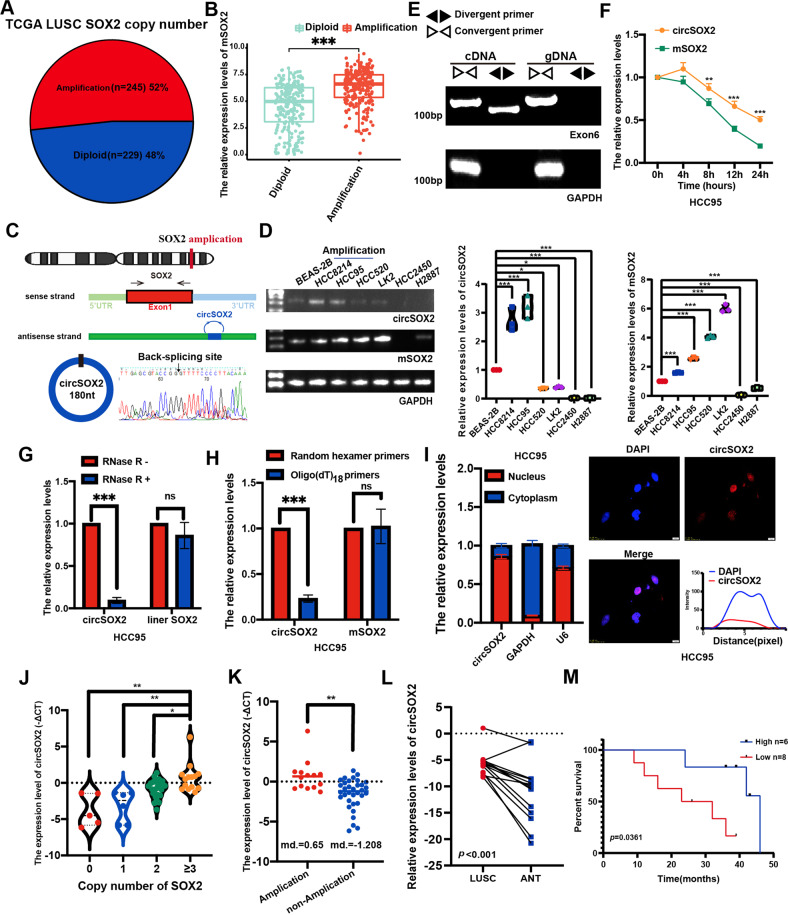
CircSOX2 could efficiently express in SOX2 amplified LUSC. **A** Pie chart of SOX2 copy number and relative proportion of LUSC in TCGA. **B** SOX2 coding transcript (mSOX2) was expressed higher than the diploid group of LUSC in TCGA. **C** The diagram illustrating the production of circSOX2. The circSOX2 was determined by Sanger sequences, and the back-splicing site has been denoted by arrows. **D** DNA gel presented the expression level of circSOX2 and mSOX2. CircSOX2 was effectively expressed in SOX2-amplificated LUSC cell lines. **E** CircSOX2 can only be amplificated by divergent primers. **F** CircSOX2 was more stable than mOX2 upon being treated with Actinomycin D. **G** CircSOX2 significantly resisted the degradation of RNase R. **H** CircSOX2 could not be reverse transcripted by poly(A)-dependent Oligo(dT)_18_ primers. **I** Nuclear and cytoplasmic fraction qRT-PCR and fluorescent in situ hybridization (FISH) revealed that circSOX2 is predominantly located in the nucleus. **J** The expression levels of circSOX2 in various SOX2 copy numbers. **K** CircSOX2 was effectively expressed in the SOX2 amplification group of LUSC. **L** The expression level of circSOX2 in LUSC and the adjacent normal tissues. **M** Kaplan–Meier curve demonstrated that SOX2-amplified LUSC patients with higher circSOX2 had better survival. ***p < 0.001, *p < 0.05, ns: no significant.

By analyzing the LUSC gene copy number profile, we identified SOX2 gene amplification in more than half of all patients with LUSC patients in The Cancer Genome Atlas (TCGA) (Fig. 1A). The expression of SOX2 messenger RNA (mRNA, mSOX2) was significantly upregulated in SOX2-amplified group in TCGA (Fig. 1B). CircSOX2 (circBase ID: hsa_circ_0122884) was the only circRNA, which derives from the SOX2 gene, annotated in circBase. CircSOX2 is derived from the reverse strand of the SOX2 gene, and the sequence is totally reverse complement to a part of the 3’UTR sequence of SOX2 coding transcript (mSOX2), which is derived from the forward strand (Fig. 1C). The divergent primer of the circSOX2 junction site was designed and verified using the Sanger sequence (Fig. 1C). We explored the expression of circSOX2 in the normal human bronchial epithelial cell line (BEAS-2B), SOX2-amplified LUSC cell line (HCC8214, HCC95), SOX2-highly expressing LUSC cell lines (HCC520 and LK2), and SOX2 gene-deficient LUSC cell lines (HCC2450 and H2887). The results indicated that circSOX2 was efficiently expressed in SOX2-amplified cells. Nevertheless, circSOX2 was weakly expressed in non-SOX2 amplified cell lines (Fig. 1D). Thus, HCC95 was selected as the highly enriched cell line, and HCC8214 was the weakly enriched one.

The qRT-PCR results indicated that circSOX2 can only be amplified using divergent primers but not a convergent primer (Fig. 1E). CircSOX2 was more stable than SOX2 coding transcript (mSOX2) following Actinomycin D treatment (Fig. 1F). CircSOX2 effectively resisted to RNase R digestion (Fig. 1G). In the reverse transcription (RT) assay, circSOX2 was not reverse-transcribed by oligo(dT)18 primers due to deficient poly(A) tail (Fig. 1H). The separation of nucleocytoplasmic RNA and fluorescence in situ hybridization (FISH) assays revealed that circSOX2 was predominantly located in the nucleus (Fig. 1I). Based on the above experiments, we supposed that circSOX2 was a nuclear circRNA originating in the SOX2 gene reverse strand.

The Taqman probes qRT-PCR was used to detect the copy number in 40 LUSC tissues. The results demonstrated that 14 patients with LUSC carried SOX2 gene amplification (Fig. 1J). Similarly, as shown in Fig. 1D, circSOX2 was significantly expressed in the amplified group, and few circSOX2 was detected in SOX2-deficient LUSC tissues (Fig. 1J). The circSOX2 level was nearly four-fold higher in the amplification group than in the non-amplification group on average (Fig. 1K). Thus, we hypothesized that the circSOX2 might be functionally expressed in LUSC cells with SOX2 gene amplification. In SOX2-amplified LUSC tissues, circSOX2 was highly expressed compared with normal adjacent tissues (Fig. 1L). The Kaplan–Meier curves indicated that patients carrying SOX2 amplification with higher circSOX2 expression had a better survival outcome (Fig. 1M).

Taken together, circSOX2 represents a potential functional nuclear circRNA associated with SOX2 amplification.

### CircSOX2 effectively suppressed the malignant progression in *SOX2*-amplified LUSC cells in vitro and in vivo

**Fig. 2:**
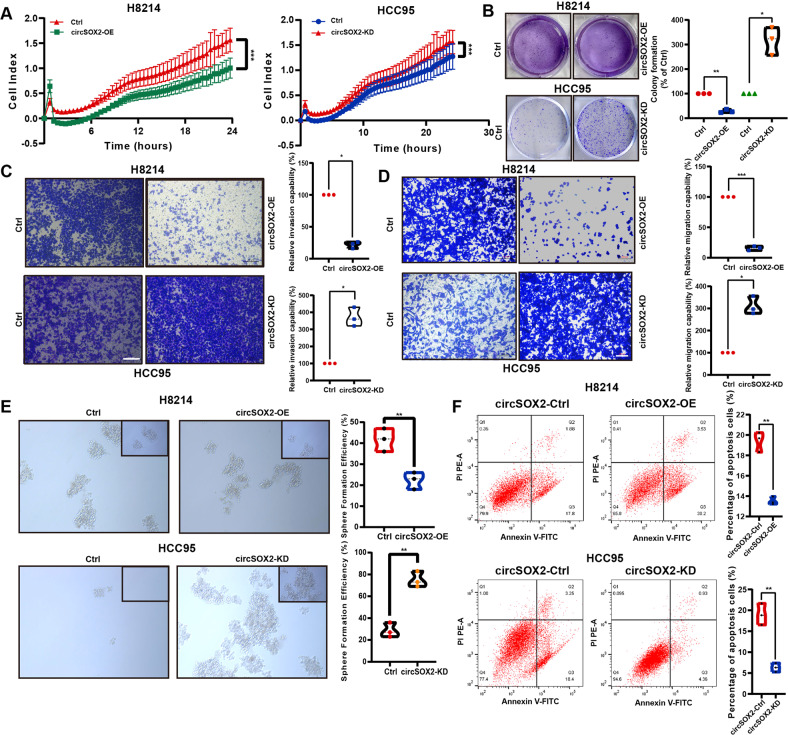
CircSOX2 suppressed the malignant progression in SOX2-amplified LUSC cells in vitro. **A** and **B** The real-time cell analysis (RTCA) assays revealed that circSOX2 suppressed the proliferation of LUSC cells. **C** and **D** Transwell and Matrigel assays indicated that circSOX2 effectively repressed the migration and invasion of LUSC cells. **E** The sphere-forming assay demonstrated that circSOX2 decreased the width and amount of LUSC sphere cells. **F** CircSOX2 overexpression suppressed the apoptosis of LUSC cells. ***p < 0.001, **p < 0.01, *p < 0.05.

Overexpressing and knockdown plasmids were efficiently designed (Fig. S1A, B). The results of real-time cell analysis (RTCA) and colony formation assay revealed that circSOX2 overexpression dramatically restricted the H8214 cell proliferation. Conversely, the knockdown of circSOX2 promoted the growth of HCC95 cells (Fig. 2A, B). Results of Transwell migration assay and Matrigel invasion assay indicated that circSOX2 suppressed the invasion and migration in LUSC cells (Fig. 2C, D). The sphere-forming assay revealed that circSOX2 significantly decreased the sphere formation rate in LUSC cells (Fig. 2E). Also, circSOX2 was involved in the apoptosis of H8214 and HCC95 cells. Overexpressed circSOX2 inhibited apoptosis in H8214 cells (Fig. 2F).

**Fig. 3:**
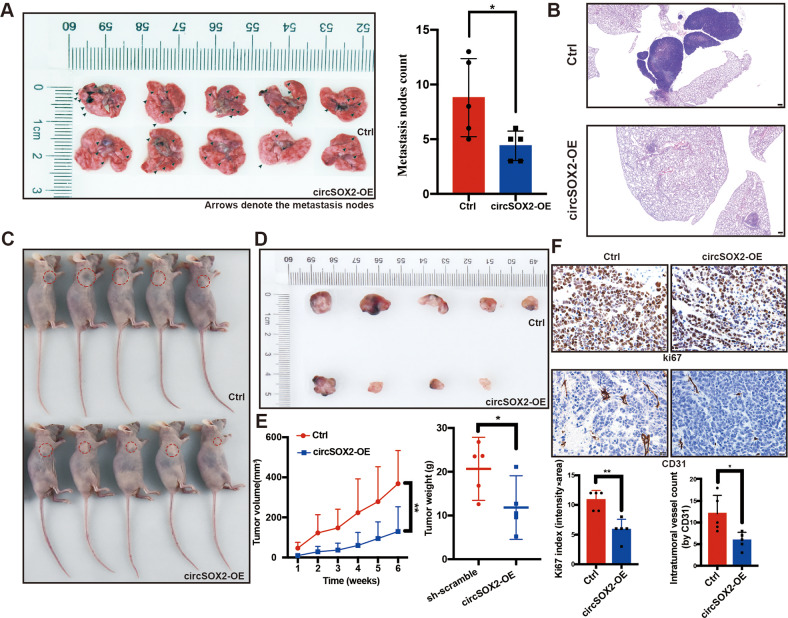
CircSOX2 suppressed the malignant progression of LUSC in vivo. **A** BALB/c nude mice were injected with cells (Ctrl and circSOX2-OE) via the tail vein and evaluated the metastasis. **B** The tumor width of the Ctrl group was bigger than the circSOX2-OE group. **C** and **D** CircSOX2 suppressed the growth of LUSC in vivo. **E** The tumor growth curves and the tumor weights of xenografts were plotted in the Ctrl and circSOX2-OE groups. **F** The images presented the immunohistochemical staining of Ki67 and CD31 in Ctrl and circSOX2-OE xenografts. **p < 0.01, *p < 0.05.

To investigate the function of circSOX2 in vivo, 6- to 8-week-old nude mice, each weighing 16–18 g, were selected. Ten mice were randomized into two equal groups: one group injected via tail vein with H8214 cells, which were stable with negative control (Ctrl) and the other using H8214 cells stable transfected with circSOX2-overexpressing plasmids. The mice were sacrificed after 6 weeks to count surface metastatic nodules. The results indicated that circSOX2 effectively suppressed the metastasis of *SOX2*-amplified LUSC cells (Fig. 3A). Hematoxylin and eosin (H&E) staining of metastatic tumors indicated that the nodules strongly expressing circSOX2 had a smaller diameter (Fig. 3B). H8214 cells with negative control (Ctrl) or circSOX2 overexpression plasmids transfected had been injected subcutaneously into two groups of nude mice, and subcutaneous tumors were obtained 6 weeks later. The tumor sizes, tumor growth curves, and weights showed that circSOX2 suppressed the growth of *SOX2*-amplified LUSC (Fig. 3C–E). The immunohistochemical staining of Ki67 and CD31 indicated that circSOX2 suppressed the growth of *SOX2*-amplified LUSC (Fig. 3F).

In conclusion, circSOX2 plays an oncosuppressive role in *SOX2*-amplified LUSC in vitro and in vivo.

### CircSOX2 acted as a self-restricted circRNA of the *SOX2* gene in *SOX2*-amplified LUSC

**Fig. 4:**
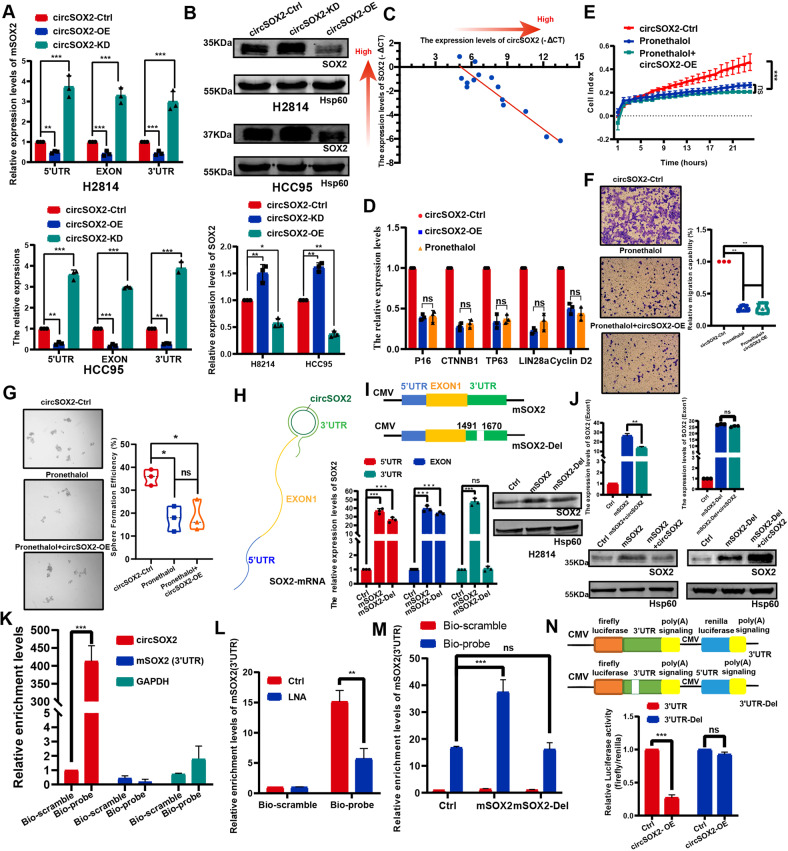
CircSOX2 was a self-restriction circRNA of the SOX2 gene in SOX2-amplified LUSC. **A** and **B** QRT-PCR and western blot assays revealed that circSOX2 suppressed the expression level of SOX2 coding transcript (mSOX2). **C** The expression of circSOX2 was negatively related to the expression level of mSOX2 in 14 SOX2-amplified LUSC tissues. **D** CircSOX2 decreased the expression of SOX2 downstream genes the same as the SOX2 transcript inhibitor, pronethalol. **E**–**G** RTCA, transwell, and sphere-forming assays indicated that the malignant progression suppression of circSOX2 was dependent on the expression of SOX2. **H** The diagram presented the hypothesis of reverse complete between circSOX2 and 3’UTR of mSOX2. **I** The overexpressed plasmids of mSOX2 and mSOX2-Del was successfully constructed and identified by qRT-PCR and western blot assays. **J** CircSOX2 could not suppress the expression of mSOX2-Del. **K** The circSOX2 probe could specially bind to circSOX2. **L** The LNA, which targeted the complete sequence of mSOX2 3’UTR, significantly decreased the binding between circSOX2 and mSOX2. **M** The mSOX2-Del plasmid could not increase the binding between circSOX2 and mSOX2 in LUSC cells. **N** The dual-luciferase reporter assays indicated that circSOX2 binds to the 3’UTR of mSOX2 dependent on the reverse complete sequences. ***p < 0.001, **p < 0.01, ns: no significant.

The localization of circRNAs partially indicates the potential regulatory mechanism in cells. Some predominantly nuclear circRNAs tend to regulate the expression of its cognate endogenous transcripts [21, 22, 27], and circSOX2 plays an essential role in SOX2-amplified LUSC cells. Thus, we investigated whether circSOX2 acts as a regulator of its cognate endogenous transcript. SOX2 gene only has a single coding transcript (ENST00000325404.3). The SOX2-coding transcript (mSOX2) contains only a single exon (Fig. 4I). We designed three dependent primers to target the 5’UTR, EXON, and 3’UTR of mSOX2, respectively. The qRT-PCR results and western blots revealed that circSOX2 restricted the expression of mSOX2 and SOX2 protein (Fig. 4A, B). In 14 SOX2-amplified LUSC tissues, the expression of circSOX2 was negatively correlated with mSOX2 (Fig. 4C). *SOX2* is a well-established oncogene in various cancers. P16, CTNNB1, TP63, LIN28a, and Cyclin D2 are the classical downstream genes of SOX2-promoting malignant progression via enhanced stemness, proliferation, invasion, and migration [7, 28, 29]. We found that circSOX2 repressed the expression of these SOX2 downstream genes, consistent with the effect of SOX2 transcription inhibitor, pronethalol [30, 31] (Fig. 4D). The RTCA, Transwell, and sphere-forming assays also indicated that circSOX2 could not repress the malignant progression further upon SOX2 transcript inhibition (Fig. 4E–G). Overall, circSOX2 suppressed the *SOX2-*amplified malignant progression of LUSC independent of *SOX2* expression.

CircSOX2 is transcribed from the reverse strand of the *SOX2* gene. Its sequence is a reverse complement to a 180 nt sequence of mSOX2 3’UTR (Fig. 1C). Thus, we investigated whether circSOX2 regulates mSOX2 by binding to the 3’UTR of mSOX2 (Fig. 4H). We constructed the wild-type *SOX2*-coding transcript overexpressing plasmid and a complement sequence-deleted plasmid (mSOX2-Del) (Fig. 4I). The 3’UTR primer of mSOX2 was designed to amplify the complement sequences. Results of QRT-PCR indicated successful construction of the mSOX2-Del plasmid (Fig. 4I). The western blot revealed that the mSOX2-Del plasmid also increased the expression of SOX2 protein, although the overexpression was less than that of the mSOX2 wild-type plasmid probably due to the decreased stability associated with incomplete 3’UTR (Fig. 4I). CircSOX2 lost the ability to decrease mSOX2 during complement sequence deletion (Fig. 4J). To explore the interaction between circSOX2 and the 3’UTR complement region, a biotin-modified oligonucleotide (bio-probe) was designed to target the back-splicing junction site, using a nonsense scramble sequence as the negative control. The pull-down assay revealed that the bio-probe was effectively bound to circSOX2 but not to mSOX2 (Fig. 4K). CircSOX2 is significantly bound to the 3’UTR region of mSOX2 (Fig. 4L). The LNA oligos, which interacted competitively with the complete sequence of the 3’UTR region, were transfected into H8214 cells. The circSOX2 probe was unable to enrich the 3’UTR region to the same degree as the control (Ctrl) (Fig. 4L). Further, the mSOX2-Del plasmid failed to enhance the levels of enriched mSOX2 3’UTR compared with the mSOX2 plasmid (Fig. 4M). Wild (3’UTR) and complement sequence-deficient mSOX2 3’UTR (3’UTR-Del) dual-luciferase reporter gene were constructed, and the circSOX2 significantly repressed the firefly luciferase in wild-type but not in the 3’UTR-Del group. The luciferase activity results further established the binding between circSOX2 and mSOX2 3’UTR (Fig. 4N).

However, circSOX2 was effectively expressed in *SOX2*-amplified LUSC cells, and circSOX2 might suppress the expression of mSOX2 by binding to its 3’UTR reverse complement sequences. We supposed that circSOX2 was a self-restricting circRNA of the *SOX2* gene in *SOX2*-amplified LUSC.

### CircSOX2 promoted mSOX2 degradation by binding AUF1 to occupy the 3’UTR of mSOX2

**Fig. 5:**
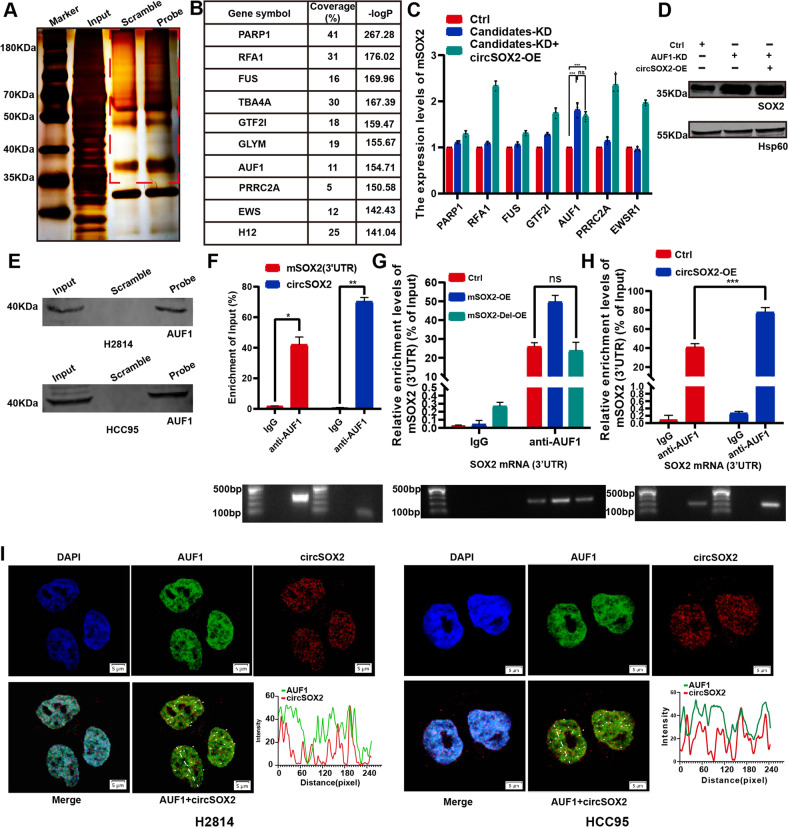
CircSOX2 promoted the mSOX2 degradation by inducing AUF1 occupied to the 3’UTR of mSOX2. **A** RNA pull-down and mass spectrometry (MS) were performed to identify proteins binding to circSOX2. **B** The candidate proteins for binding to circSOX2. **C** and **D** CircSOX2 was unable to regulate mSOX2 expression upon AUF1 knockdown. **E** The pull-down products identified that circSOX2 bind to AUF1. **F** RIP qRT-PCR confirmed that AUF1 binds to circSOX2 and mSOX2. **G** The mSOX2-Del overexpression could not increase the interaction between circSOX2 and mSOX2 3’UTR. **H** CircSOX2 overexpression increased the interaction between circSOX2 and the 3’UTR of mSOX2. **I** FISH indicated the co-localization of AUF1 and circSOX2 in H2814 and HCC95 cells. ***p < 0.001, **p < 0.01, ns: no significant.

CircRNA was recognized as an inducer guiding sponge protein to the target oligonucleotide [32, 33]. To explore whether circSOX2 induces the binding of some proteins to the 3’UTR of mSOX2, the precipitates of pull-down were separated via SDS–PAGE, and silver staining was performed. The dissimilar bands were analyzed via liquid chromatography–tandem mass spectrometry (LC–MS/MS), and the top 10 proteins were selected as the candidates based on the coverage (%) (Fig. 5A, B). Based on the literature review, seven of the proteins were associated with carcinogenesis. We found that circSOX2 could not rescue mSOX2 upregulation which was induced by AUF1 knockdown (Figs. 5C, D and S1C). AUF1 is a nuclear protein with canonical roles in regulating the stability and translation of mRNA targets via recognition of AU-rich sequences within 3’ untranslated regions of mRNA [34]. The pull-down concomitant western blot and RNA immunoprecipitation (RIP) assays confirmed the interaction between circSOX2 and AUF1 (Fig. 5E, F). Further, the RIP assay demonstrated that AUF1 interacted with mSOX2 3’UTR, while mSOX2-Del plasmid did not increase this enrichment compared with wild-type mSOX2 plasmid (Fig. 5G). To determine whether circSOX2 was an inducer of AUF1, the RIP assay was performed. The results indicated that circSOX2 overexpression significantly increased the levels of mSOX2 3’UTR enrichment in AUF1 (Fig. 5H). AUF1 is specifically bound to AU-rich elements (AREs) to degrade the mRNAs [34]. The 3’ UTR region of mSOX2 near the complete sequences carries consecutive “AUUUA” pentamers (Fig. S1D). The RNA FISH-immunofluorescence analysis revealed the binding between circSOX2 and AUF1 in the nucleus of HCC95 and H8214 cells (Fig. 5I).

**Fig. 6:**
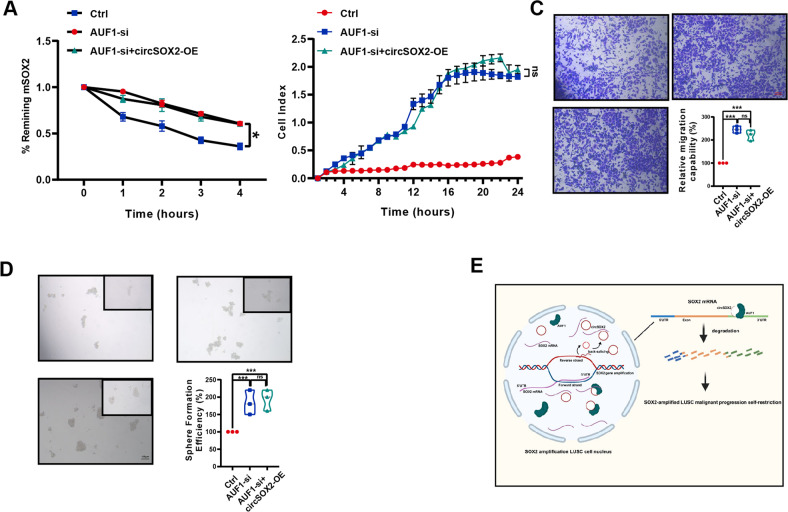
CircSOX2 suppressed the expression of mSOX2 dependent on AUF1. **A** CircSOX2 was unable to effectively degrade mSOX2 in the absence of AUF1. **B**–**D** RTCA, transwell, and sphere-forming assays indicated that circSOX2 expression could not suppress the malignant progression upon AUF1 knockdown. **E** Illustration of circSOX2 involvement in LUSC. ***p < 0.001, *p < 0.05, ns: no significant.

Actinomycin D analysis revealed that AUF1 was required for circSOX2-mediated degradation of mSOX2 (Fig. 6A). In a further study, we found that circSOX2 could not rescue the AUF1 knockdown-induced growth, metastasis, and sphere-forming suppression partially (Fig. 6B–D). Taken together, circSOX2 induced the degradation of mSOX2 in SOX2-amplified LUSC by sponging and guiding AUF1 to occupy the 3’UTR of mSOX2.

## Discussion

In this study, circSOX2 was identified as a functional self-restricted circRNA in *SOX2*-amplified LUSC. Interestingly, circSOX2 was effectively expressed in *SOX2*-amplified LUSC cell lines and tissues. However, it functionally suppressed the proliferation, invasion, migration, and sphere formation in *SOX2*-amplified LUSC in vitro and in vivo. Mechanistically, circSOX2 serves as an AUF1 guide. Since mSOX2 and circSOX2 were transcribed on the forward and reverse strands of *SOX2*, respectively, the sequence of circSOX2 was reverse-complement to a portion of the 3’UTR of mSOX2. CircSOX2-bound AUF1 occupied the 3’UTR, which recognized the AU-rich elements and initiated the degradation of mSOX2. We concluded that circSOX2 was a functional nucleic acid drug candidate for SOX2-amplified LUSC.

In some studies, circRNAs might regulate specific transcripts. The sequences of circRNAs were reverse complement to specific mRNAs occasionally. The circRNA binds to the mRNA based on the complementary pairing principle. CircZNF609 interacted with CKAP5 mRNA across its junction site, serving to guide HuR binding and maintaining normal mitotic progression [35]. Several circRNAs may compete with liner mRNAs for protein binding. CircHomer1 decreased the translation of its cognate endogenous transcription by competing for binding to HuR to improve the orbitofrontal cortex and regulate reverse learning [36]. CircFAM120A bound competitively to the m6A reader protein, IGF2BP2, and with FAM120A mRNA to maintain the translation of liner mRNA [37]. In our study, circSOX2 was derived from the reverse strand of the *SOX2* gene, which merely corresponds to a portion of the 3’UTR of the *SOX2*-coding transcript. Thus, we hypothesized that circSOX2 interacted with the 3’UTR of the *SOX2*-coding transcript. Further experiments identified this binding.

Stable circRNAs originate in pre-mRNAs with low immunogenicity in vivo. The development of the nucleic acid drug, which was based on RNA circle-based technologies, elicited substantial interest. CircRNAs might represent noncoding aptamers to sponge disease-related miRNAs or proteins. An artificially designed circRNA carried four miR-122-binding sites to promote exonucleolytic degradation of hepatitis C virus (HCV). It was more efficient than the single miR-122 complete LNA-oligonucleotides, Miravirsen [38]. AAV delivered circularized circ-INSR mimics to sponge the SSBP1, resulting in cardioprotection in vivo [39]. The circularly engineered antisense RNA is more stable than the linear one. A circRNA targeting the conserved 5’UTR of SARS-CoV-2 reduced viral proliferation by ~90%. The circular antisense oligonucleotides were stabler than ASOs modified with 20-O-methyl (20-OMe) or 20-O-methoxyethyl(20-MOE) [40]. CircRNAs with IRES showed higher translation duration compared with liner transcription [41]. CircRNA SARS-CoV-2 vaccines encoded the receptor-binding domain of the spike protein constructed to induce antibody synthesis. These studies expanded the application of circRNAs in disease therapy. In our study, circSOX2 was a self-splicing endogenous circRNA targeting *SOX2* in *SOX2*-amplified LUSC. Thus, circSOX2 carries a huge translational potential for patients with *SOX2*-amplified LUSC. CircSOX2 can be used as a nucleic acid drug to restrict the *SOX2* expression dramatically and abolish the driver gene amplification in *SOX2*-amplified LUSC.

## Conclusion

In this study, we reported circSOX2, a novel circRNA that was effectively expressed in *SOX2*-amplified LUSC. By inducing mSOX2 degradation, circSOX2 inhibited cell proliferation, metastasis, and sphere formation. CircSOX2 interacted with and guided AUF1 to the 3’UTR of mSOX2, which was dependent on the reverse complement sequence. Thus, circSOX2 represents a self-splicing circRNA of the *SOX2*-amplified gene in LUSC (Fig. 6E).

## Materials and methods

### Tissues collection

The experiment conformed to regulatory requirements and was approved by the Ethics Committee of the First Affiliated Hospital of Soochow University. Informed consent forms were signed by all of the patients. Forty human lung squamous cell carcinoma (LUSC) and adjacent normal tissue (ANT) pairs were obtained from the First Affiliated Hospital of Soochow University between 2017 and 2020. (Nanjing, China). It was determined, with the help of experienced pathologists, that LUSC tissues and coupled ANT are accurate diagnostic indicators.

### Cell culture

The Chinese Academy of Sciences Cell Bank supplied the normal human bronchial epithelial cell line (BEAS-2B, RRID: CVCL 0168), the HCC95 cells (RRID: CVCL 5137), the H2814 cells (RRID: CVCL 6898), the HCC520 cells (RRID: CVCL 1566), the LK2 cells (RRID: CVCL W132), the HCC2450 cells (RRID: CVCL 5133), BEAS-2B cells were grown in fetal bovine plasma(Gibco, Grand Island, USA)-supplemented RPMI-1640 media (Keygen Biotech, China). Other types of cancer cells (HCC95, HCC520, LK2, HCC2450, and H2887) were grown in RPMI-1640 medium (Keygen Biotech, China) with 10% fetal bovine plasma (Gibco, Grand Island, USA). Cells were maintained in a humidified 37 °C incubator with 5% CO_2_. The cells have been validated within the last 3 years by Guangzhou Biotech Corp. and no mycoplasma has been detected.

### TCGA

We downloaded data of TCGA LUSC copy number variation (CNV) (GISTIC Annotation) from UCSC Xena Browser (accessed on 19 July 2021), which included 474 samples. The copy number values and their discrete indicator are as following: serious deletion = −2; deletion = −1; no change = 0; amplification = 1; high amplification = 2.

### Copy number variation analysis

DNA was isolated from LUSC tissues using QIAamp DNA Mini Kit, according to the manufacturer’s protocol (Qiagen). Copy number variation was evaluated on 20 ng of genomic DNA. Quantitative real-time polymerase chain reaction (PCR) TaqMan Copy Number Assays were performed using three probes targeting the SOX2 gene (GeneScript, Nanjing, China). qRT-PCR was performed using a TaqMan copy number assay kit (ThermoFisher Scientific) according to the manufacturer’s protocols. RNase P was co-amplified and used as an internal control (TaqMan Copy Number Reference Assay). Real-time data were collected by step one Plus the Real-Time PCR method (ABI) and its associated instruments. Copy Numbers ranging from 1.5 to 2.5 were predicted as CNV = 2. Three independent assays were performed for each sample to confirm the results. The probe and primers have been listed in Supplementary Table 1.

### Apoptosis flow cytometry

Cells were stained with propidium iodide (PI) and FITC Annexin V (#556,570; BD Biosciences, San Diego, CA, USA) for 15 min at 25 °C to determine the apoptotic rate. Using a FACSCalibur flow cytometer, we studied early apoptotic cells (FITC Annexin V+/PI) and late apoptotic cells (FITC Annexin V+/PI+) (BD Biosciences, San Diego, CA, USA).

### In vivo animal model and growth, metastasis assays

We randomly split 20 female BALB/c nude mice (weighing 18–22 g) into four groups. To administer the H2814-Ctrl and H2814-circSOX2-OE cells, a solution of 4 × 10^5^ cells in 200 μl saline was used. At 6 weeks post-injection, mice were sacrificed and analyzed for the presence of lung metastases and H&E staining. The H2814-Ctrl and H2814-circSOX2-OE cells were used to form subcutaneous xenografts in the unilateral axillary of nude mice. Every week, a caliper was used to measure the size of the growing tumor. After 6 weeks, the mice were sacrificed, and the weight of the subcutaneous xenograft tumors was recorded. Animal experimentation complied with all requirements of the Chinese State Food and Drug Administration. There was no intentional excluding or including of any animals in the sorting process, which was limited to categorizing them by therapy.

### Statistics

All statistical analyses used SPSS 25.0. Qualitative variables were evaluated using chi-square or fisher’s exact test. Normal data are used for the Student’s *t*-test. Abnormal distribution variables were tested nonparametrically. Comparing groups using ANOVA. Pearson correlation analysis was used. Means SD shows the results (SD). All statistical tests were two-sided, and *p* < 0.05 was significant.

## Supplementary information

Reproducibility checklist:

Supplementary Figure1

Supplementary Materials and Methods

Supplementary Table1

Original Data File

## Data Availability

The datasets generated or analysed in the current study are available from the corresponding author upon reasonable request.
