# Supplementary material for: Self-restricted circular RNA circSOX2 suppressed the malignant progression in SOX2-amplified LUSC
Source: Cell Death Dis. 2022 Oct 15;13(10):873. doi: 10.1038/s41419-022-05288-5 (PMC9568965; doi:10.1038/s41419-022-05288-5)
Supplement: Supplementary file 2 — Supplementary Figure1 [file 41419_2022_5288_MOESM2_ESM.pdf]

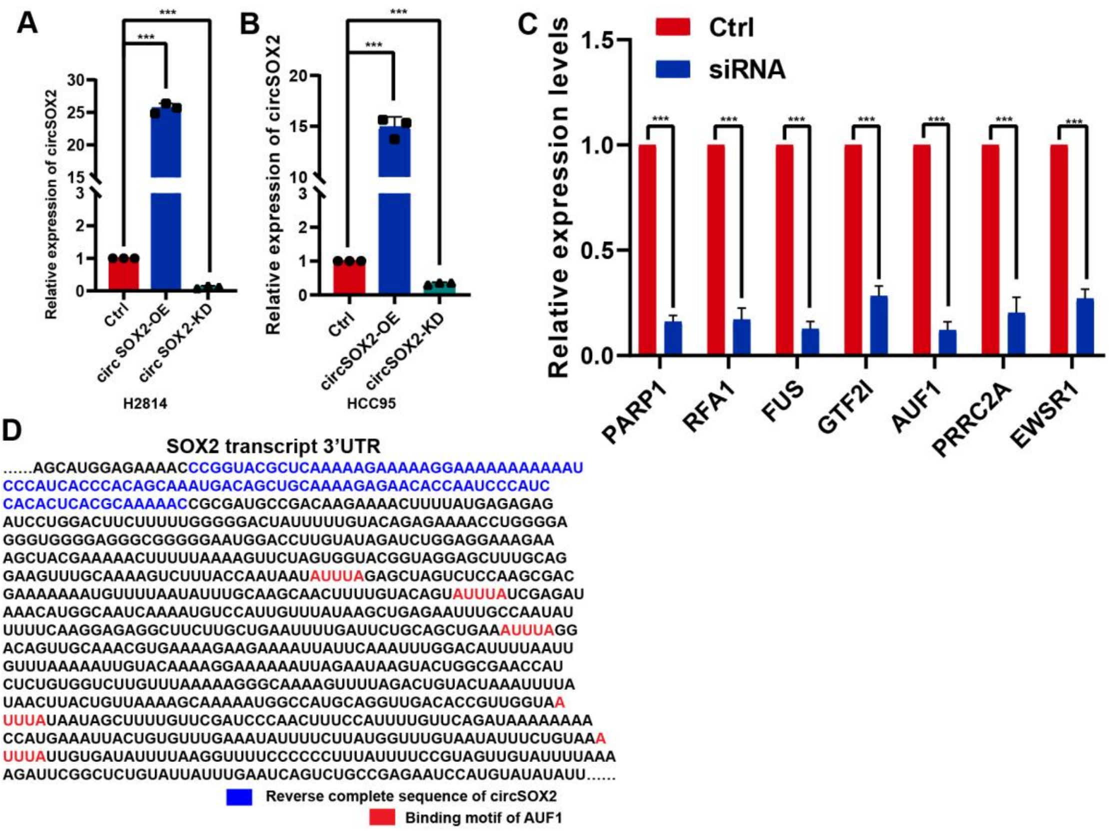

Supplementary Figure 1 (A-B) The efficiency of circSOX2 overexpression and knockdown plasmid in H2814 and HCC95 cells. (C) The efficiency of binding protein candidate knockdown. (D) The “AUUUA” motif sites in 3’UTR of mSOX2.
