# Supplementary material for: Self-restricted circular RNA circSOX2 suppressed the malignant progression in SOX2-amplified LUSC
Source: Cell Death Dis. 2022 Oct 15;13(10):873. doi: 10.1038/s41419-022-05288-5 (PMC9568965; doi:10.1038/s41419-022-05288-5)
Supplement: Supplementary file 3 — Supplementary Materials and Methods [file 41419_2022_5288_MOESM3_ESM.docx]

**Supplementary Materials and Methods**

**Quantitative PCR and RNase R treatment**

Isolating RNA from cells has been accomplished using Trizol reagent (Takara, Dalian, China). The cell fragmentation isolation kit was utilized for nuclear and cytoplasmic RNA and protein extraction (Fisher Scientific, Vilnius, Lithuania). The RNase R solution was added to 1 g of total RNA that had been incubated at 37°C for 30 minutes, and the reaction was then allowed to continue while the mixture was boiled (Geneseed, Shanghai, China). Then, reverse transcription (Takara) or oligo (dT) (Qiagen, Hilden, Germany) Quantitative PCR using SYBR Green master mix (Takara) has been carried out. CircSOX2 transcript levels were determined using back-splicing primers. We designed convergent primers that amplify 5’UTR, EXON1, and 3’UTR of SOX2 transcript. The amplification and analysis were performed using step one Plus the Real-Time PCR method (ABI, Foster City, CA) and its associated instruments. 2^-ΔΔCT^ was used to quantify the level of expression. Purification of the circIMMP2L SOX2 transcript and GAPDH amplification product was performed using a PCR purification kit (Qiagen). The primers were listed in the Supplementary Table1.

**Actinomycin D assay**

Five wells of 5×104 HCC95 cells were seeded in 24-well plates. After 24 hours, cells were treated with actinomycin D (2g/ml, Abcam, Cambridge, UK) and collected in 0h, 4h, 8h, 12h, and 24h, respectively. qRT-PCR was used to compare circSOX2 and SOX2 transcript RNA levels to the 0h group.

**RNA-Fluorescence in situ hybridization assay and Fluorescence immunocytochemical staining**

With the guidance of an RNA-FISH kit (GenePharma, China), we were able to conduct RNA-Fluorescence in situ hybridization (FISH) experiments as directed by the manufacturer. GenePharma Company (Suzhou, China) designed and produced a Cy3-labeled antisense probe targeting the circSOX2 junction. The supplementary Table1 provides the probe sequence. Paraformaldehyde at a concentration of 4% was used to fix HCC95 and H2814 cells. Cells were pre-hybridized in 1x PBS/0.5% Triton X-100, blocked, then hybridized overnight in hybridization buffer containing a Cy3-labeled probe at 37 °C. After that, AUF1-specific antibodies (1:200 dilution) were added to the cells and they were left in a 4 °C incubator overnight. A concentration of 300 nmol/L of DAPI was used to stain the cells. The probe and antibody have been listed in Supplementary Table1.

**Over-expression or knockdown of genes**

Human SOX2 linear sequence (contained 5’UTR, EXON1, and 3’UTR) and SOX2 liner mutation sequences inserted into plasmid vector pcDNA 3.1 (Hanbio, shanghai, China). (Hanbio, shanghai, China). For construction of plasmids encoding circSOX2, the full length of human circSOX2 cDNA was amplified by PCR using the following primers: circSOX2-forward: taatgactttttttttatacttcagGTTTTCTCTGTACAAAAATAGT and circSOX2-reverse: cctaattcttttccttgcttcttacCCGGTACGCTCAAAAAGAAAAAG. Then, the circSOX2 sequence was ligated into pcDNA3.1-circRNA vector after restriction digests (Hanbio). The small interfering RNA (siRNA) of PAPR1, RFA1, FUS, GTF2I, AUF1, PRRCW2A, and EWSR1 were provided by RiboBio (Guangzhou, China). The target sequences were supplied in Supplementary Table1. shRNAs against circSOX2 and corresponding control were obtained from the Sangon Biotech (Shanghai, China). The target sequences were supplied in Supplementary Table1.The transient transfection of the shRNA or the overexpressing plasmids were performed using the Lipofectamine 3000 kit (Invitrogen, Carlsbad, CA) according to manufacturer’s instructions, and the transient transfection of siRNA were performed using the Lipofectamine iMax kit (Invitrogen) according to manufacturer’s instructions.

**Real-time cell analysis (RTCA)**

To monitor the cellular proli formation we utilized the “xCELLigence” system (Roche Applied Sciences and ACEA Biosciences) including 16-well E-plates for continuous and label-free detection. After setting up the program, 50 μL of the culture medium per well was dropped into the E-plate to plot the baseline, followed by seeding 8000 H2814 and HCC95 cells in each well. The cells were placed at room temperature for 30 min to attach to the E-plate before subsequent detection. Cells were incubated at 37℃ and 5% CO2 and Cell Index (CI) was monitored every 30 min for the duration of the experiment.

**Colony formation**

A total of 1×10^3^ cells were seeded into each well of a 6-well cell culture plate, and the media was changed out every 3 days. At the end of 14 days, the colonies were counted after being fixed in 4% paraformaldehyde for 10 minutes, stained for 15 minutes with a 0.05% crystal violet solution, and finally counted.

**Transwell and Matrigel assay**

In a serum-free medium, 4×10^4^ cells were seeded in the upper transwell assay chambers with 8μm pore filters (Millipore, Massachusetts, USA) for the migration experiment. For the invasion assay, 4×10^4^ cells were seeded in a serum-free medium into the upper matrigel assay chambers with a matrigel-coated membrane (Corning, Massachusetts, USA). The medium in the lower chamber included 10% FBS as chemokine. After incubation at 37°C for 24 hours for migration and 48 hours for invasion, non-migrating or non-invading cells were removed softly, and cells that migrated and invaded to the bottom of the membrane were fixed with 4% paraformaldehyde, stained with crystal violet solution for 30 minutes, and observed under a microscope at 100 magnification.

**Sphere formation assay**

Ultra-low Attachment six-well plates were used to culture H2814 and HCC95 cells (Corning, NY, USA). To grow the cells, 2×10^5^ were cultured in serum-free PRMI-1640 medium supplemented with EGF, hFGF (Peprotech, USA), insulin, and penicillin/streptomycin (Gibco). Clones of spheroids were fixated and identified using a light stereomicroscope (Olympus, Tokyo, Japan).

**Western blot assay**

Briefly, the total protein of cells was extracted using RIPA (Thermo Fisher Scientific, Waltham, USA) with a cocktail of proteinase and phosphatase inhibitors (Thermo Fisher Scientific) according to its protocol. Equal quantities of protein lysates were resolved by SDS-PAGE gels and then transferred on a PVDF membrane (Millipore, Massachusetts, USA). The membranes were hybridized at room temperature with a secondary antibody for 1 hour after incubation with the primary antibody at 4°C overnight. Odyssey XF Operator was used to visualize blots (Lincoln, Nebraska, USA). The antibody information is listed in Supplementary Table1.

**Dual-luciferase reporter assay**

5×10^4^cells were plated into a 96-well plate and cultivated for 24 hours. According to the protocol included in the Dual-Luciferase Reporter Assay Kit, a luciferase assay was performed using two different luciferases (Vazyme, Nanjing, China). We lysed the cells and investigated for luciferase 48 hours after transfection. Normalization of firefly luciferase activity to Renilla luciferase activity was performed. Information is presented as a percentage of control cell luciferase activity.

**RNA Pull-down and mass spectrometry (MS) analysis**

Biotin-labeled RNA probes for circSOX2 and scramble were produced by GenePharma Company (Suzhou, China). The RNA pull-down test utilized a Biotin-Pull-Down Kit for Protein-Protein Interactions (Thermo Fisher Scientific). Lysis buffer containing 2x10^7^ cells was kept cold and incubated on ice for 30 min. Biotinylated probes were treated with streptavidin-coated magnetic beads for 30 minutes at room temperature. Lysis was then combined with the beads probe complex at 4 ° C for two hours. Those proteins that had been bound to the hermetically packed beads were eventually released. Mass spectrometry (MS) analysis, silver staining, and a western blot assay were used to study the proteins. We included the probe sequences in Table S1.

**RNA immunoprecipitation assay (RIP)**

This RIP experiment was performed using the Magna RIPTM RNA-Binding Protein Immunoprecipitation Kit (Millipore) according to the manufacturer's instructions. 2×10^7^ cells were placed in lysis buffer and kept on ice for 10 minutes. At room temperature, 5μg of antibody was incubated with magnetic beads. Overnight at 4 °C, tissue lysates were exposed to the bead. The immunoprecipitated RNAs were isolated after being treated with proteinase K. QRT-PCR was used to determine how abundant circSOX2 and mSOX2 were. The RIP procedure utilized antibodies specific for AUF1 and IgG. The Co-precipitated RNA was tracked using quantitative real-time PCR. Primers and antibodies were provided in the Supplementary Table1.
