# Supplementary material for: Self-restricted circular RNA circSOX2 suppressed the malignant progression in SOX2-amplified LUSC
Source: Cell Death Dis. 2022 Oct 15;13(10):873. doi: 10.1038/s41419-022-05288-5 (PMC9568965; doi:10.1038/s41419-022-05288-5)
Supplement: Supplementary file 4 — Supplementary Table1 [file 41419_2022_5288_MOESM4_ESM.docx]

**Supplementary Table1**

**QRT-PCR Primers**

SOX2 5’UTR

Forward TTGCTGCCTCTTTAAGACTAGGA

Reverse CTGGGGCTCAAACTTCTCTC

SOX2 EXON1

Forward CATGAAGGAGCACCCGGATT

Reverse TAACTGTCCATGCGCTGGTT

SOX2 3’UTR

Forward AGCATGGAGAAAACCCGGTA

Reverse TTTTGCGTGAGTGTGGATGG

GAPDH

Forward ACAACTTTGGTATCGTGGAAGG

Reverse GCCATCACGCCACAGTTTC

U6

Forward primer: CTCGCTTCGGCAGCACA

Reverse primer: AACGCTTCACGAATTTGCG

PAPR1

Forward primer: TGGAAAAGTCCCACACTGGTA

Reverse primer: AAGCTCAGAGAACCCATCCAC

RFA1

Forward primer: GGGGATACAAACATAAAGCCCA

Reverse primer: CGATAACGCGGCGGACTATT

FUS

Forward primer: ATGGCCTCAAACGATTATACCCA

Reverse primer: GTAACTCTGCTGTCCGTAGGG

AUF1

Forward primer: GCGTGGGTTCTGCTTTATTACC

Reverse primer: TTGCTGATATTGTTCCTTCGACA

GTF2I

Forward primer: TTGTCGTCGGAACTGAAAGAG

Reverse primer: CGATTTGCCTGGGTTGTAGAT

PRRC2A

Forward primer: GAGTTCCGATGCCTCAACCG

Reverse primer: CCAGGACTTTACCCCGCTT

EWSR1

Forward primer: ATGGCGTCCACGGATTACAG

Reverse primer: GTGCATATCCTTGAGTGGGCT

P16

Forward primer: GATCCAGGTGGGTAGAAGGTC

Reverse primer: CCCCTGCAAACTTCGTCCT

CTNNB1

Forward primer: AAAGCGGCTGTTAGTCACTGG

Reverse primer: CGAGTCATTGCATACTGTCCAT

P63

Forward primer: GGTTGGCAAAATCCTGGAG

Reverse primer: GGTTCGTGTACTGTGGCTCA

LIN28A

Forward primer: AGCGCAGATCAAAAGGAGACA

Reverse primer: CCTCTCGAAAGTAGGTTGGCT

Cyclin 2D

Forward primer: GCTGCGAAGTGGAAACCATC

Reverse primer: CCTCCTTCTGCACACATTTGAA

P21

Forward primer: TGTCCGTCAGAACCCATGC

Reverse primer: AAAGTCGAAGTTCCATCGCTC

**Taqman primers and probe of SOX2**

Primer: Forward AATGCCTTCATGGTGTGGTC

Reverse GCTTAGCCTCGTCGATGAAC

Probe: 5’Fam-ATCAGCAAGCGCCTGGGCGC-3’Tamra

**Antibodies**

| **Gene symbol** | **Cat. number** | **Supplier** |
| --- | --- | --- |
| SOX2 | ab92494 | Abcam |
| AUF1 | ab259895 | Abcam |
| Hsp60 | ab190828 | Abcam |
| GAPDH | 51332S | CST |

**Small inhibitor**

Pronethalol

cat. Number: HY-B1238

Supplier: MCE MedChemExpress

**Small hairpin RNA (shRNA) Target Sequence**

CircSOX2-KD (sh-circSOX2)

ACCGGGTTTTCTCTGTACA

Ctrl

5′-GTGCGAGGGGGTTGTAATCTT-3′

**Small interfering RNA (siRNA) Target sequence**

si-PAPR1

5′-GAGGAAGGTATCAACAAATTT-3′

si-RFA1

5′-AACTGGTTGACGAAAGTG-3′

si-FUS

5′-GGACAGCAGCAAAGCTATA-3′

si-GTF2I

5′-AAGTTACTCAGCCAAGAACGA-3′

si-AUF1

5′-GAGCCGGTTAAAAAAATTT-3′

si-PRRC2A

5ʹ-CCCAACACCAGAGACAGAACCTGAA-3’

si-EWSR1

5’-TGCATTGACTACCAG ATTTAT-3′

Ctrl

5′-GTGCGAGGGGGTTGTAATCTT-3′
