# Supplementary figures and images for: Self-restricted circular RNA circSOX2 suppressed the malignant progression in SOX2-amplified LUSC
Source: Cell Death Dis. 2022 Oct 15;13(10):873. doi: 10.1038/s41419-022-05288-5 (PMC9568965; doi:10.1038/s41419-022-05288-5)

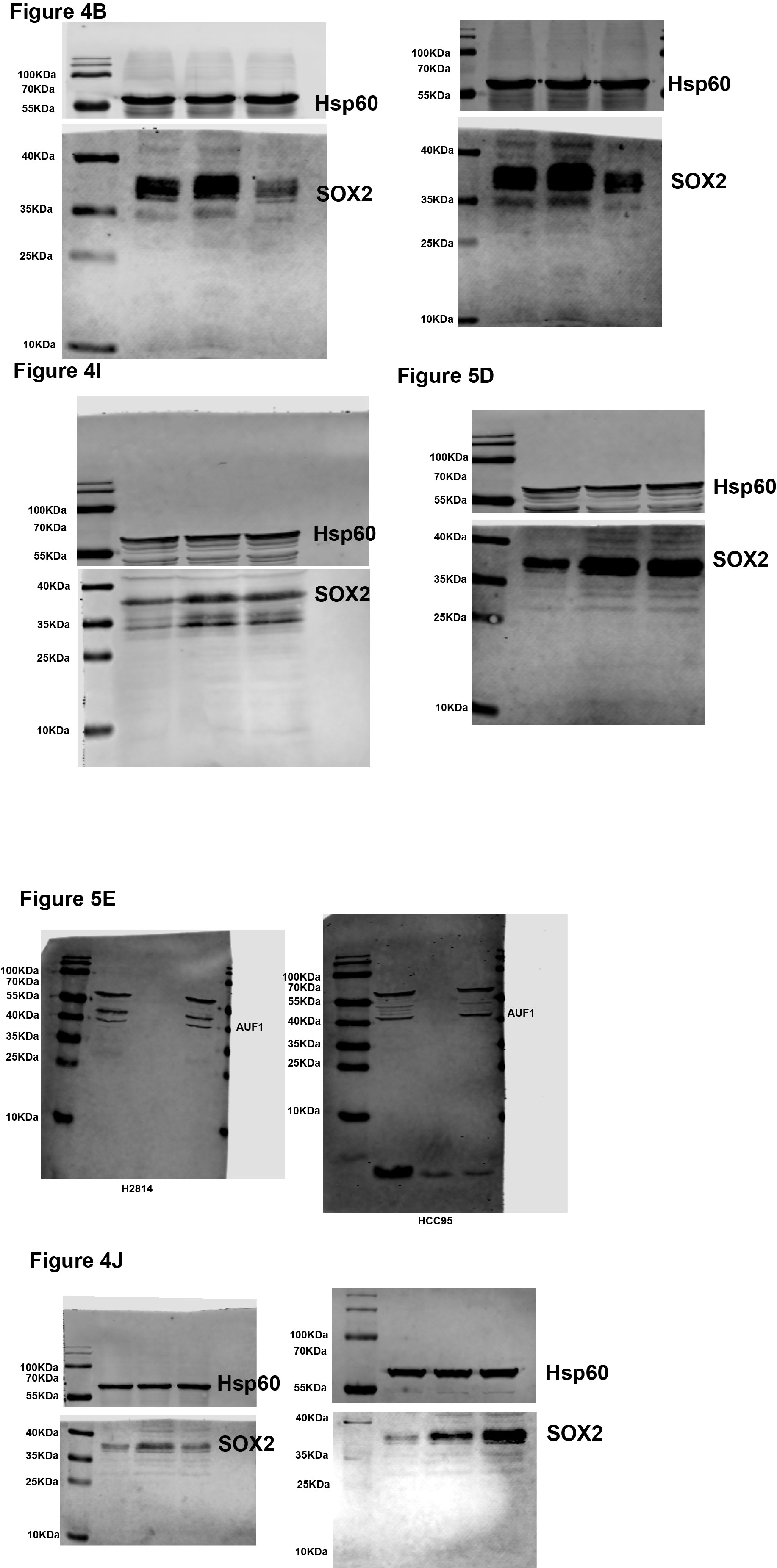

Supplement: Supplementary file 5 — Original Data File [file 41419_2022_5288_MOESM5_ESM.jpg]
